# Progress in Expression Pattern and Molecular Regulation Mechanism of LncRNA in Bovine Mastitis

**DOI:** 10.3390/ani12091059

**Published:** 2022-04-20

**Authors:** Li Jia, Jinpeng Wang, Zhuoma Luoreng, Xingping Wang, Dawei Wei, Jian Yang, Qichao Hu, Yun Ma

**Affiliations:** 1 School of Agriculture, Ningxia University, Yinchuan 750021, China; jialiesther@163.com (L.J.); 13309402775@126.com (J.W.); weidaweiwdw@163.com (D.W.); yangjian9603@163.com (J.Y.); hqc13629560035@126.com (Q.H.); mayun@nxu.edu.cn (Y.M.); 2 Key Laboratory of Ruminant Molecular Cell Breeding, Ningxia Hui Autonomous Region, Yinchuan 750021, China

**Keywords:** lncRNA, cow, mastitis, expression pattern, regulatory mechanism

## Abstract

**Simple Summary:**

Bovine mastitis is an inflammatory disease of the mammary glands that causes serious harm to cow health and huge economic losses. Susceptibility or resistance to mastitis in individual cows is mainly determined by genetic factors, including coding genes and non-coding genes. Long non-coding RNAs (lncRNAs) are non-coding RNA molecules with a length of more than 200 nucleotides (nt) that have recently been discovered. They can regulate a variety of diseases of humans and animals, especially the immune response and inflammatory disease process. This paper reviews the role of long non-coding RNA (lncRNA) in inflammatory diseases, emphasizes on the latest research progress of lncRNA expression and the molecular regulatory mechanism in bovine mastitis, and looks forward to the research and application prospect of lncRNA in bovine mastitis, intending to provide a reference for scientific researchers to systematically understand this research field.

**Abstract:**

Bovine mastitis is an inflammatory disease caused by pathogenic microbial infection, trauma, or other factors. Its morbidity is high, and it is difficult to cure, causing great harm to the health of cows and the safety of dairy products. Susceptibility or resistance to mastitis in individual cows is mainly determined by genetic factors, including coding genes and non-coding genes. Long non-coding RNAs (lncRNAs) are a class of endogenous non-coding RNA molecules with a length of more than 200 nucleotides (nt) that have recently been discovered. They can regulate the immune response of humans and animals on three levels (transcription, epigenetic modification, and post-transcription), and are widely involved in the pathological process of inflammatory diseases. Over the past few years, extensive findings revealed basic roles of lncRNAs in inflammation, especially bovine mastitis. This paper reviews the expression pattern and mechanism of long non-coding RNA (lncRNA) in inflammatory diseases, emphasizes on the latest research progress of the lncRNA expression pattern and molecular regulatory mechanism in bovine mastitis, analyzes the molecular regulatory network of differentially expressed lncRNAs, and looks forward to the research and application prospect of lncRNA in bovine mastitis, laying a foundation for molecular breeding and the biological therapy of bovine mastitis.

## 1. Introduction

Bovine mastitis is an inflammatory disease of the mammary glands caused by pathogenic microbial infections, trauma, or other stimuli. Its morbidity is high and difficult to cure, causing serious harm to cow health and dairy product safety [1]. Every year, the disease causes huge economic losses worldwide due to factors such as treatment costs, labor, veterinary services, a reduction in milk production, and the need to discard inferior milk [2]. Therefore, bovine mastitis is an urgent problem to be solved. It is necessary to further study the roles and molecular mechanisms of bovine mastitis.

Long non-coding RNA (lncRNA) is an endogenous non-coding RNA molecule with a length of more than 200 nucleotides (nt) [3] that plays an important role in the transcriptional and epigenetic regulation of human and animal genes [4,5], and has become a research focus of gene expression regulation in recent years. In the organism’s immune response, long non-coding RNAs (lncRNAs) regulate the occurrence and development progress of various inflammatory diseases, including bovine mastitis. In this paper, we review the expression patterns and mechanisms of action of lncRNAs, with emphasis on the latest research on the role of lncRNAs in bovine mastitis. The main objective of this review is to better understand bovine mastitis from the perspective of lncRNAs, and to explain the classification, expression, and potential role of lncRNAs in regulating bovine mastitis.

## 2. Classification of LncRNA

**Figure 1:**
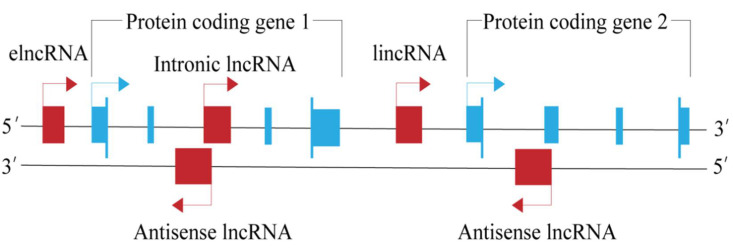
Classification of lncRNAs.

In 2002, scientists first proposed the concept of lncRNA after the large-scale sequencing of a full-length mouse complementary DNA (cDNA) library [6]. At present, scholars have a clearer understanding of the origin, size, and conservative characteristics of lncRNAs [7]. However, there is still no unified standard for their classification. Currently, the more commonly used method is to classify lncRNAs according to the relative position of the lncRNA and the encoding gene [8]. According to this method, lncRNAs can be divided into four types, including long intergenic lncRNAs (lincRNAs), enhancer lncRNAs (elncRNAs), intronic lncRNAs, and antisense lncRNA (Table 1, Figure 1). This reflects the function of lncRNAs to some extent, and can also help researchers to intuitively understand lncRNAs.

## 3. Mechanism of Action of LncRNAs

**Figure 2:**
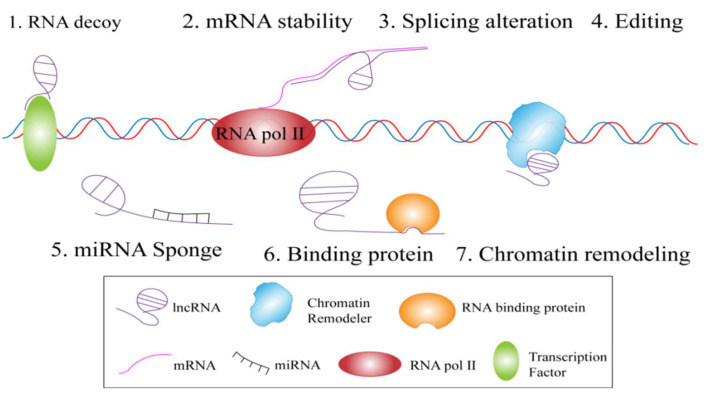
Mechanism of action of lncRNAs.

The mechanism of action of lncRNAs is complex and diverse, involving multiple biological processes, such as messenger RNA (mRNA) synthesis, mRNA processing, and protein expression [17]. Furthermore, the special structure and subcellular localization of lncRNAs enable them to interact with a variety of molecules [18,19,20] and to play a biological role through transcription regulation, epigenetic modification, and post-transcriptional levels (Figure 2) [21,22,23].

### 3.1. Role of LncRNAs at the Transcriptional Level

Studies have shown that lncRNAs can act as both cis-acting and trans-acting regulators of gene expression [24,25]. For example, lincRNA-Cox2 can regulate the expression of its adjacent gene prostaglandin-endoperoxide synthase (*Ptgs2*) through an elncRNA mechanism, and can also regulate the expression of innate immune genes interleukin-5 (*IL-5*), leukemia inhibitory factor (*LIF*), and interleukin-17 (*IL-17*) by a trans-acting mechanism, thereby affecting the immune process [26,27,28,29]. As transcriptional regulators of adjacent genes, the elncRNAs have a strong function in regulating gene expression [30,31,32]. In addition, lncRNAs can exert signal transduction and induction functions. For example, lncRNA X inactive specific transcript (XIST) can be transcribed and wrapped on the inactivated X chromosome, thereby inhibiting the gene expression level of the entire chromosome [33]. Moreover, lncRNAs can serve as a scaffold or bridge for chromatin modifications to play a regulatory role, such as linc-RAM, which, when directly bound to myogenic differentiation (MyoD), promotes the assembly of a MyoD-Baf60c-Brg1 complex, thereby enhancing myogenic differentiation [34].

### 3.2. Role of LncRNAs in Epigenetic Modification

The biological processes in the body cannot be fully understood from a genetic perspective alone, but are affected by both genetic and environmental factors. Thus, epigenetic modifications affect growth and development, metabolism, and disease states through DNA methylation, chromatin remodeling, or histone modification, without changing the DNA sequence [35,36,37]. XIST, the first discovered lncRNA, can recruit chromatin remodeling complexes PRC2 and participate in the amino acid methylation modification of histone H3, which eventually results in the inactivation of the X chromosome and mediates gender-related diseases [38]. In addition, lncRNA ecCEBPA has been found to act on the transferase DNA methyltransferase 1 (*DNMT1*), thereby changing the methylation of the CCAAT/enhancer binding protein alpha (*CEBPA*) gene and increasing its expression level, which may make DNA methylation modification a novel therapeutic target for human diseases [39].

### 3.3. Post-Transcriptional Regulation through LncRNAs

LncRNAs can affect the initiation and elongation cycle of protein translation, leading to either the stimulation or inhibition of mRNA translation. Studies have shown that lncRNAs can form double-stranded RNA complexes with target mRNA through complementary base pairing, thereby affecting the splicing, translation, and degradation of mRNAs [28]. LncRNA-p21 can inhibit mRNA translation by stimulating the translation suppressor Rck to complementarily bind to the target mRNA [40]. In addition, lncRNA can also serve as a molecular sponge for miRNAs, competitively adsorbing miRNAs to prevent them from binding to and inhibiting the translation of their target mRNAs, thereby promoting the expression of target genes [41,42,43]. For instance, studies have shown that lncRNA MEG3 plays an anti-inflammatory role in ankylosing spondylitis by inhibiting the expression of inflammatory factors through the sponge adsorption of miR-146a [43].

## 4. Research Progress on the Role of LncRNAs in Bovine Mastitis

The genetic factors and environmental factors jointly affect the occurrence and development of bovine mastitis [44]. Among them, genetic factors are directly related to the susceptibility and disease resistance of mastitis. Therefore, an in-depth analysis of the molecular mechanism of bovine mastitis on a genetic level can lay the foundation for the breeding of new lines of cows that will be resistant to mastitis. LncRNAs are novel regulators discovered in recent years that play a role in regulating the body’s immune system. As mentioned above, the differential expression of lncRNAs can regulate the inflammatory disease process in humans and model animals. However, the conservation of lncRNA sequences between different species is low, and the sequence of target genes for regulation also varies among different species, which makes the results of the research on model animals and humans difficult to apply directly to cows [23,45]. Therefore, an in-depth study of the mechanism of action of lncRNAs in bovine mastitis is still needed. Compared with humans or model animals, such as mice and rats, studies on lncRNAs related to mastitis in cows have been lagging behind. So far, these concern only the screening of differentially expressed lncRNAs and the study of the molecular mechanism of action of a few specific lncRNAs.

### 4.1. Screening of Differentially Expressed LncRNAs in Bovine Mastitis

High-throughput sequencing technology has revealed a high correlation between lncRNA expression and various diseases, including mastitis, which has opened up new avenues to analyze the pathogenic and disease-resistant mechanisms of bovine mastitis. In recent years, in general, lncRNAs with differential expression (including specific expression) or elevated expression in inflammatory diseases were selected as the targets of research into the molecular mechanism of disease. At present, only a few studies have reported the screening of differentially expressed lncRNAs for bovine mastitis using RNA-seq technology. Tong et al. [46] found four up-regulated and five down-regulated lncRNAs in milk exosomes from cows before and after infection with *S. aureus*. It is worth mentioning that these differentially expressed lncRNAs are closely associated with the *S. aureus* invasion of mammary epithelial cells, and may be related to the way cells communicate with each other and the defense mechanisms or pathways involved in inflammatory responses [46]. In addition, 184 lncRNAs were identified from five RNA-seq datasets from bovine mammary glands, of which, TCONS_00071212 was located in the clinical mastitis quantitative trait locus region [47]. In addition, another TCONS_0007853 could be involved in the development of the bovine mammary gland via the Rap1 signaling pathway and MAPK signaling pathway [47]. Wang et al. [48] identified 53 differentially expressed lncRNAs in primary bovine mammary alveolar cells (MAC-T) induced by *E. coli* and *S. aureus* using high-throughput sequencing, of which, four lncRNAs, i.e., TCONS_00048953, TCONS_00048966, TCONS_00049002, and TCONS_00025496, were located in the clinical mastitis-related quantitative trait locus region. Again, Ozdemir and Altun [49] identified 392 novel lncRNAs in bovine mammary gland tissues. Furthermore, 57 lncRNAs (of which, 19 were novel lncRNAs) were differentially expressed between normal mammary gland tissues and mammary gland tissues infected by *Mycoplasma bovis*, and these were involved in the regulation of important biological pathways, including PI3K-Akt, NF-κB, and mTOR [49], implying functions in cancer, immunity, and apoptosis. In addition, 21 differentially expressed lncRNAs (including 13 up-regulated and 8 down-regulated) were found in *S. aureus*-infected MAC-T cells, and a bioinformatics analysis revealed that the above differentially expressed lncRNAs might be involve in NF-κB and TNF signaling pathways [50]. More recently, in the transcriptional profiling of exosomes, Chen et al. [51] found 19 differentially expressed lncRNAs from *S. aureus*-treated and untreated MAC-T cells, and these lncRNAs participated in inflammation-related signal pathways (i.e., the Notch, TNF, and NF-κB signal pathway). At the same time, Wang et al. [52] also found 112 differentially expressed lncRNAs in lipopolysaccharides (LPS)-treated bovine mammary epithelial cells (bMECs) after 0, 6, and 12 hours that might be involved in the regulation of Notch, NF-κB, mTOR, MAPK, PI3K-Ak, and other inflammation-related signal pathways. In brief, a comprehensive comparison of the differential expression of lncRNAs in the literature revealed that the differential expression levels of various lncRNAs in different studies were not the same, speculating that lncRNAs may be sensitive regulators of bovine mastitis, and that their expression levels may be related to the individual status of the cow, the type of infectious bacteria, the infective dose, and the time of infection. At the same time, different types of lncRNAs may be differentially expressed at different stages of infection by pathogenic microorganisms in order to participate in the regulation of the entire range and intensity of the immunological defense mechanisms.

### 4.2. Molecular Function of LncRNAs in Bovine Mastitis

Compared with the current research on the regulation of human and mouse inflammatory diseases by lncRNAs, the studies on the regulatory mechanism of bovine mastitis by lncRNAs are still lagging behind. So far, only a few lncRNAs (including lncRNA H19, lncRNA TUB, lncRNA XIST, and LRRC75A-AS1) have been studied with respect to their role in bovine mastitis.

#### 4.2.1. LncRNA H19

**Figure 3:**
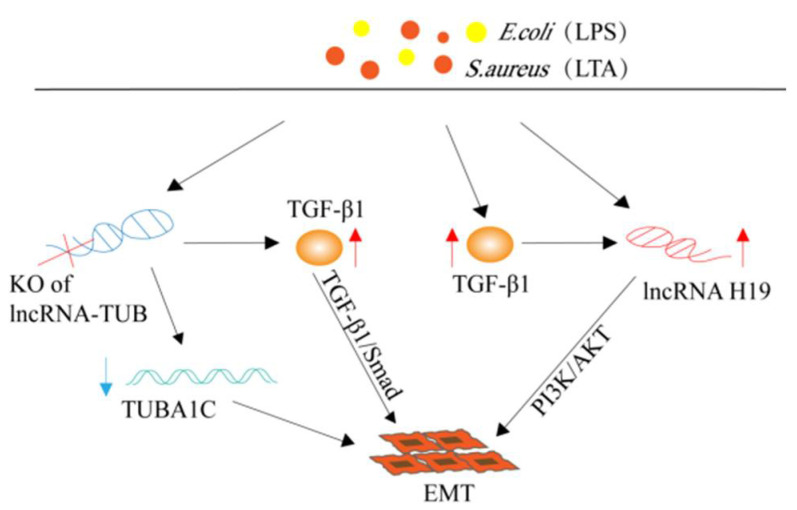
The effect of lncRNA-H19 and lncRNA-TUB on the epithelial–mesenchymal transition of inflammatory mammary epithelial cells.

LncRNA H19 is a previously discovered lncRNA [53] whose biological functions include roles in cardiovascular diseases [54] and abdominal aortic aneurysms [55], as well as other human diseases [56,57]. In the mammary gland inflammation of cows, Yang et al. [58] found that the expression levels of H19 in bovine mammary gland tissue with mastitis and lipoteichoic acid (LTA) or LPS-induced MAC-T cells were up-regulated. In addition, it has been shown that the overexpression of TGF-β1 in MAC-T cells caused up-regulation of the expression of lncRNA H19 through the PI3K-AKT signal pathway. Subsequently, an increased expression of H19 can induce epithelial–mesenchymal transition (EMT), which would result in a decreased milk production in dairy cows (Figure 3) [58]. During chronic inflammation, bovine mammary epithelial cells undergo EMT and turn into muscle fiber cells, thereby secreting a large amount of the extracellular matrix (ECM), causing its excessive accumulation [59]. At the same time, the continuous secretion of cytokines and chemokines by inflammatory cells deepens the inflammatory infiltration, leading to the formation of fibrosis [60]. These studies revealed the regulatory mechanism of H19 in the process of bovine mammary fibrosis. It will be interesting to see whether research analyzing the molecular mechanism of bovine mastitis and the relationship between mastitis and fibrosis will lead to a breakthrough in its treatment. In addition, Li et al. [61] found that lncRNA H19 can promote the proliferation of MAC-T cells and the expression of β-casein and tight junction (TJ)-related proteins (claudin-1, occludin, and ZO-1). Moreover, lncRNA H19 can also inhibit the adhesion of *S. aureus* to cells, which is critical in blocking infection by these pathogenic bacteria and in promoting the effective recovery of mammary gland tissues of diseased cows. At the same time, the overexpression of lncRNA H19 results in the activation of NF-κB inflammatory pathways in MAC-T cells and the release of inflammatory factors, including TNF-α, IL-6, CXCL2, and CCL5. This results in a rapid clearing of the pathogenic factors and promotes the return of the body to a steady state [61]. In summary, lncRNA H19 plays an active role in inhibiting bacterial infection and restoring the normal function of inflamed tissue.

#### 4.2.2. LncRNA-TUB

Bovine mastitis often causes a series of pathological changes in the body. Similar to H19, lncRNA-TUB can also promote EMT in MAC-T cells. Wang et al. [48] identified a new type of lncRNA from an *E. coli* and *S. aureus*-induced MAC-T cell inflammatory model, named lncRNA-TUB. That study found that lncRNA-TUB was up-regulated in the above mastitis cell model [48]. The knock-out of lncRNA-TUB resulted in a reduction in the proliferation and migration ability of MAC-T cells and in the secretion of β-casein, and resulted in the up-regulation of IL-1β and IL-8 and down-regulation of TNF-α and IL-6 [48]. In addition, the deletion of lncRNA-TUB resulted in the down-regulation of its target gene TUBA1C and the up-regulation of TGF-β1, causing EMT in MAC-T cells [48]. It is worth noticing that the increased secretion of TGF-β1 activated the TGF-β1/Smad pathway and promoted EMT (Figure 3) [62]. As mentioned above, mastitis caused MAC-T cells to undergo EMT and to display ECM accumulation, and excessive ECM will aggravate the inflammatory response, which, in turn, induces the secretion of chemokines and inflammatory factors, further aggravating EMT in a vicious cycle, then ultimately causing fibrosis of mammary tissue [60]. Therefore, the discovery of lncRNA-TUB can provide a useful reference for studying bovine mastitis and fibrosis caused by mastitis.

#### 4.2.3. LncRNA XIST

**Figure 4:**
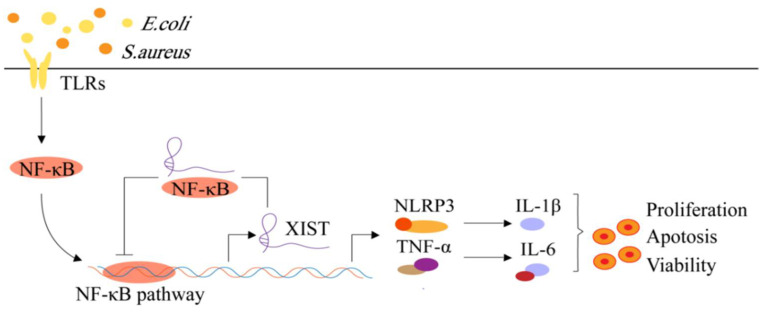
The molecular mechanism of XIST negative feedback regulates bovine mastitis through the NF-κB / NLRP3 inflammatory body pathway.

XIST is a 17 kb lncRNA transcribed from the inactivated X chromosome that has been studied widely [63,64]. XIST is involved in the regulation of cancer and gender-related diseases, although its molecular mechanism is very complex [33,64]. Ma et al. [65] have shown that an *S. aureus* or *E. coli*-induced inflammatory response in MAC-T cells resulted in the rapid activation of the intracellular NF-κB signaling pathway, thereby promoting the production of NLRP3 inflammasome and pro-inflammatory cytokines. However, the activated NF-κB pathway also promoted a significant increase in the expression of lncRNA XIST. The highly expressed lncRNA XIST inhibited the activation of the NF-κB inflammatory pathway by a negative feedback mechanism, which, in turn, inhibited the formation of the NLRP3 inflammasome and the secretion of pro-inflammatory cells factors (TNF-α, IL-1β, and IL-6), resulting in the alleviation of the cellular inflammatory response (Figure 4) [65]. In addition, it was also found that, under inflammatory conditions, lncRNA XIST can regulate the NF-κB inflammation pathway through negative feedback to inhibit apoptosis, promote cell proliferation, and maintain cell viability (Figure 4) [65]. Thus, XIST can mediate the inflammatory process of bovine MAC-T cells through the NF-κB/NLRP3 inflammasome axis. Bovine mastitis is a defense response of the body against foreign pathogens that can lead to the up-regulation or down-regulation of lncRNA expression. However, excessive inflammation can cause damage to the body. Therefore, some lncRNAs positively or negatively regulate the inflammatory process to maintain the body’s homeostasis [66,67,68]. Moreover, lncRNA MALAT1 has been found to produce a similar regulatory mechanism in the inflammatory response of human monocyte macrophages [69].

#### 4.2.4. LRRC75A-AS1

**Figure 5:**
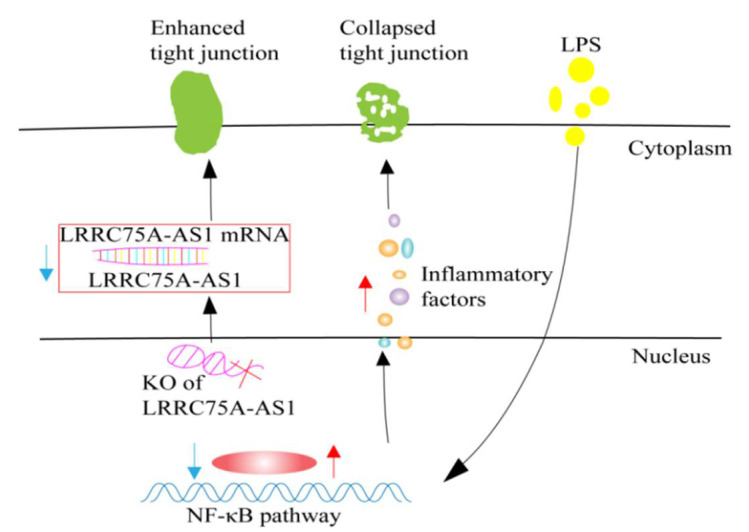
Hypothesis that LRRC75A-AS1 regulates the immune response of MAC-T cells.

Studies have shown that *E. coli* or LPS-induced MAC-T cells can activate the NF-κB pathway and produce inflammatory factors. A large number of inflammatory factors can destroy the structure of tight junctions (TJs), and can promote the infectious ability of pathogenic microorganisms, thus aggravating the cellular inflammatory response, forming a positive feedback loop [70]. LRRC75A-AS1 is a lncRNA of approximately 4kb, transcribed from the antisense strand of leucine-rich repeat-containing protein 75A (LRRC75A), which can bind to the coding sequence of LRRC75A mRNA to protect LRRC75A from nuclease degradation [71]. It has been shown that the expression levels of LRRC75A-AS1 in *E. coli*-treated MAC-T cells, *E. coli*-treated primary mammary epithelial cells, LPS-treated MAC-T cells, and mammary tissue with mastitis were all significantly decreased compared with control groups [71]. The down-regulation of LRRC75A-AS1 resulted in a decrease in the expression level of LRRC75A, which, in turn, increased the expression levels of TJ structure-related proteins (claudin-1, occludin, and ZO-1), thereby protecting the TJ structure from destruction. As a result, the cell monolayer permeability was reduced, which ultimately reduced the adhesion and invasion of *S. aureus* (Figure 5) [71]. At the same time, knocking out LRRC75A-AS1 results in a decrease in the expression of nuclear phosphorylated p65, alleviating the inflammation induced by *E. coli* [71]. Therefore, it was speculated that, during the onset of bovine mastitis caused by *E. coli* or *S. aureus*, the mammary tissue is protected from excessive inflammatory damage by the down-regulation of LRRC75A-AS1 expression.

#### 4.2.5. Other LncRNAs

In recent years, a small number of lncRNAs have been discovered that can regulate the proliferation and vitality of bMECs [61,65]. Studies have shown that NONBTAT017009.2 is a key molecule in the miR-21-3p regulatory network that can specifically bind to miR-21-3p and indirectly regulate the expression of insulin-like growth-factor-binding protein 5 (*IGFBP5*), resulting in a decreased vitality and proliferation of bMECs and reduced lactation performance of dairy cows [72]. Yang et al. [73] found that two lncRNAs (TCONS_00000352 and TCONS_00040268) had significant inhibitory effects on the proliferation and viability of bMECs. At the same time, they also found that two other lncRNAs (TCONS_00015196 and TCONS_00087966) could improve the proliferation and vitality of bMECs through the sponge adsorption of miR-221. Moreover, it was found that, after interfering with these four lncRNAs, the expression of cell-proliferation-related genes (cyclin D1 (*CCND1*), cyclin B1 (*CCNB1*), cyclin-dependent kinase inhibitor 1A (*CDKN1A*), cyclin-dependent kinase inhibitor 1B (*CDKN1B*), cyclin E1 (*CCNE1*), and cyclin-dependent kinase 2 (*CDK2*)) had changed in varying degrees [73]. The above studies explored the regulatory role of lncRNAs in bovine mammary gland development and the lactation cycle. However, whether these lncRNAs were involved in bovine mastitis or in the repair of mammary tissue damage in dairy cows remains to be determined.

Additionally, of most interest was the regulatory network of lncRNA and target genes in bovine mastitis. Tucker et al. [74] found that certain lncRNAs (including NONBTAT027932.1 and XR_003029725.1) participated in the lipopolysaccharide-mediated signaling pathway to regulate bovine mastitis, which may act as biomarkers for molecular breeding and biotherapy.

## 5. Conclusions and Future Perspectives

Bovine mastitis is difficult to cure since the molecular mechanism of its pathogenesis and development is very complex. As key regulators of the expression of immune-related genes, lncRNAs have been shown to play an important role in mastitis in cows. In recent years, high-throughput sequencing and other related technologies have been used to screen and identify a large number of lncRNAs implicated in bovine mastitis. In other words, scientific research on the role of lncRNAs in the susceptibility and resistance to bovine mastitis shows huge potential. However, studies on the molecular mechanism of the regulation of the inflammation of bovine mammary glands by lncRNAs have been highly lacking. So far only a few (less than 4%) of differentially expressed lncRNAs, such as lncRNA H19, lncRNA-TUB, lncRNA XIST, and LRRC75A-AS1, have been functionally studied, and the regulation mechanisms of many more differentially expressed lncRNAs on their target genes and mammary inflammation need to be further investigated, which can help to clarify the molecular regulatory network of lncRNA in bovine mastitis. Systematically elucidating the molecular network of differentially expressed lncRNAs implicated in bovine mastitis can provide new research thoughts for the development of molecular diagnosis and treatment technology for bovine mastitis and molecular breeding of dairy cows resistant to mastitis.

## Figures and Tables

**Table 1:** Classification of lncRNAs.

Category	Definition	References	Examples
long intergenic lncRNA (lincRNA)	LincRNAs are the longest RNA transcripts, are located between annotated protein-coding genes, and are at least 1 kb away from the nearest protein-coding genes	[9]	lincRNA BCILN25 [10]
enhancer lncRNA (elncRNA)	ElncRNAs are closely related to enhancer–promoter interactions near the coding gene regulation	[11]	CRED9 [12]
intronic lncRNA	Intronic lncRNAs are located in the intron of the coding gene	[13]	intronic lncRNA ANRASSF1 [14]
antisense lncRNA	Antisense lncRNAs are transcribed from the antisense strand and overlap part of the sense strand	[15]	lncRNA FOXC2-AS1 [16]

## Data Availability

Not applicable.

## References

[1] Puerto M.A., Shepley E., Cue R.I., Warner D., Dubuc J., Vasseur E. (2021). The hidden cost of disease: I. Impact of the first incidence of mastitis on production and economic indicators of primiparous dairy cows. J. Dairy Sci..

[2] Ashraf A., Imran M. (2020). Causes, types, etiological agents, prevalence, diagnosis, treatment, prevention, effects on human health and future aspects of bovine mastitis. Anim. Health Res. Rev..

[3] Ren Z., Yu Y., Chen C., Yang D., Ding T., Zhu L., Deng J., Xu Z. (2021). The triangle relationship between long noncoding RNA, RIG-I-like receptor signaling pathway, and glycolysis. Front. Microbiol..

[4] Dykes I.M., Emanueli C. (2017). Transcriptional and post-transcriptional gene regulation by long non-coding RNA. Genom. Proteomics Bioinform..

[5] Yao Z.T., Yang Y.M., Sun M.M., He Y., Liao L., Chen K.S., Li B. (2022). New insights into the interplay between long non-coding RNAs and RNA-binding proteins in cancer. Cancer Commun. (Lond.).

[6] Okazaki Y., Furuno M., Kasukawa T., Adachi J., Bono H., Kondo S., Nikaido I., Osato N., Saito R., Suzuki H. (2002). Analysis of the mouse transcriptome based on functional annotation of 60,770 full-length cDNAs. Nature.

[7] Quinn J.J., Chang H.Y. (2016). Unique features of long non-coding RNA biogenesis and function. Nat. Rev. Genet..

[8] Kung J.T., Colognori D., Lee J.T. (2013). Long noncoding RNAs: Past, present, and future. Genetics.

[9] Ulitsky I., Bartel D.P. (2013). LincRNAs: Genomics, evolution, and mechanisms. Cell.

[10] Xu S., Liu H., Wan L., Zhang W., Wang Q., Zhang S., Shang S., Zhang Y., Pang D. (2019). The MS-lincRNA landscape reveals a novel lincRNA BCLIN25 that contributes to tumorigenesis by upregulating *ERBB2* expression via epigenetic modification and RNA-RNA interactions in breast cancer. Cell Death Dis..

[11] Ding M., Liu Y., Liao X., Zhan H., Liu Y., Huang W. (2018). Enhancer RNAs (eRNAs): New insights into gene transcription and disease treatment. J. Cancer.

[12] Setten R.L., Chomchan P., Epps E.W., Burnett J.C., Rossi J.J. (2021). CRED9: A differentially expressed elncRNA regulates expression of transcription factor CEBPA. RNA.

[13] Ayupe A.C., Tahira A.C., Camargo L., Beckedorff F.C., Verjovski-Almeida S., Reis E.M. (2015). Global analysis of biogenesis, stability and sub-cellular localization of lncRNAs mapping to intragenic regions of the human genome. RNA Biol..

[14] Beckedorff F.C., Ayupe A.C., Crocci-Souza R., Amaral M.S., Nakaya H.I., Soltys D.T., Menck C.F., Reis E.M., Verjovski-Almeida S. (2013). The intronic long noncoding RNA ANRASSF1 recruits PRC2 to the *RASSF1A* promoter, reducing the expression of *RASSF1A* and increasing cell proliferation. PLoS Genet..

[15] Wery M., Gautier C., Descrimes M., Yoda M., Migeot V., Hermand D., Morillon A. (2018). Bases of antisense lncRNA-associated regulation of gene expression in fission yeast. PLoS Genet..

[16] Zhang C.L., Zhu K.P., Ma X.L. (2017). Antisense lncRNA FOXC2-AS1 promotes doxorubicin resistance in osteosarcoma by increasing the expression of FOXC2. Cancer Lett..

[17] Zhang K., Shi Z.M., Chang Y.N., Hu Z.M., Qi H.X., Hong W. (2014). The ways of action of long non-coding RNAs in cytoplasm and nucleus. Gene.

[18] Su Z.D., Huang Y., Zhang Z.Y., Zhao Y.W., Wang D., Chen W., Chou K.C., Lin H. (2018). iLoc-lncRNA: Predict the subcellular location of lncRNAs by incorporating octamer composition into general PseKNC. Bioinformaticss.

[19] Zhang Y., Huang Y.X., Jin X., Chen J., Peng L., Wang D.L., Li Y., Yao X.Y., Liao J.Y., He J.H. (2021). Overexpression of lncRNAs with endogenous lengths and functions using a lncRNA delivery system based on transposon. J. Nanobiotechnol..

[20] Huang Y., Qiao Y., Zhao Y., Li Y., Yuan J., Zhou J., Sun H., Wang H. (2021). Large scale RNA-binding proteins/LncRNAs interaction analysis to uncover lncRNA nuclear localization mechanisms. Brief Bioinform..

[21] Yao R.W., Wang Y., Chen L.L. (2019). Cellular functions of long noncoding RNAs. Nat. Cell Biol..

[22] Kazimierczyk M., Kasprowicz M.K., Kasprzyk M.E., Wrzesinski J. (2020). Human long noncoding RNA interactome: Detection, characterization and function. Int. J. Mol. Sci..

[23] Roberts T.C., Morris K.V., Weinberg M.S. (2014). Perspectives on the mechanism of transcriptional regulation by long non-coding RNAs. Epigenetics.

[24] Singh A.P., Luo H., Matur M., Eshelman M.A., Hamamoto K., Sharma A., Lesperance J., Huang S. (2022). A coordinated function of lncRNA HOTTIP and miRNA-196b underpinning leukemogenesis by targeting FAS signaling. Oncogene.

[25] Mahmoudi Y., Hossein Pourhanifeh M., Rajabi A., Bahabadi Z.R., Mohammadi A.H., Rahimian N., Hamblin M.R., Mirzaei H. (2021). Roles of non-coding RNAs and angiogenesis in glioblastoma. Front. Cell Dev. Biol..

[26] Chen H., Du G., Song X., Li L. (2017). Non-coding transcripts from enhancers: New insights into enhancer activity and gene expression regulation. Genom. Proteom. Bioinform..

[27] Villegas V.E., Zaphiropoulos P.G. (2015). Neighboring gene regulation by antisense long non-coding RNAs. Int. J. Mol. Sci..

[28] Engreitz J.M., Haines J.E., Perez E.M., Munson G., Chen J., Kane M., McDonel P.E., Guttman M., Lander E.S. (2016). Local regulation of gene expression by lncRNA promoters, transcription and splicing. Nature.

[29] Elling R., Robinson E.K., Shapleigh B., Liapis S.C., Covarrubias S., Katzman S., Groff A.F., Jiang Z., Agarwal S., Motwani M. (2018). Genetic models reveal cis and trans immune-regulatory activities for lincRNA-Cox2. Cell Rep..

[30] Lam M.T., Li W., Rosenfeld M.G., Glass C.K. (2014). Enhancer RNAs and regulated transcriptional programs. Trends Biochem. Sci..

[31] Font-Cunill B., Arnes L., Ferrer J., Sussel L., Beucher A. (2018). Long non-coding RNAs as local regulators of pancreatic islet transcription factor genes. Front. Genet..

[32] Paralkar V.R., Taborda C.C., Huang P., Yao Y., Kossenkov A.V., Prasad R., Luan J., Davies J.O., Hughes J.R., Hardison R.C. (2016). Unlinking an lncRNA from its associated cis element. Mol. Cell..

[33] Pontier D.B., Gribnau J. (2011). Xist regulation and function explored. Hum. Genet..

[34] Yu X., Zhang Y., Li T., Ma Z., Jia H., Chen Q., Zhao Y., Zhai L., Zhong R., Li C. (2017). Long non-coding RNA linc-RAM enhances myogenic differentiation by interacting with MyoD. Nat. Commun..

[35] Dates C.R., Tollefsbol T.O. (2018). Transforming cancer epigenetics using nutritive approaches and noncoding RNAs. Curr. Cancer Drug Targets.

[36] Long H., Yin H., Wang L., Gershwin M.E., Lu Q. (2016). The critical role of epigenetics in systemic lupus erythematosus and autoimmunity. J. Autoimmun..

[37] Wei J.W., Huang K., Yang C., Kang C.S. (2017). Non-coding RNAs as regulators in epigenetics (review). Oncol. Rep..

[38] Jeon Y., Lee J.T. (2011). YY1 tethers Xist RNA to the inactive X nucleation center. Cell.

[39] Di Ruscio A., Ebralidze A.K., Benoukraf T., Amabile G., Goff L.A., Terragni J., Figueroa M.E., De Figueiredo P.L., Alberich-Jorda M., Zhang P. (2013). DNMT1-interacting RNAs block gene-specific DNA methylation. Nature.

[40] Yoon J.H., Abdelmohsen K., Srikantan S., Yang X., Martindale J.L., De S., Huarte M., Zhan M., Becker K.G., Gorospe M. (2012). LincRNA-p21 suppresses target mRNA translation. Mol. Cell..

[41] Li Y., Chen D., Gao X., Li X., Shi G. (2017). LncRNA NEAT1 regulates cell viability and invasion in esophageal squamous cell carcinoma through the miR-129/CTBP2 axis. Dis. Markers.

[42] Tay Y., Rinn J., Pandolfi P.P. (2014). The multilayered complexity of ceRNA crosstalk and competition. Nature.

[43] Li Y., Zhang S., Zhang C., Wang M. (2020). LncRNA MEG3 inhibits the inflammatory response of ankylosing spondylitis by targeting miR-146a. Mol. Cell Biochem..

[44] Nani J.P., Raschia M.A., Carignano H., Poli M.A., Calvinho L.F., Amadio A.F. (2015). Single nucleotide polymorphisms in candidate genes and their relation with somatic cell scores in Argentinean dairy cattle. J. Appl. Genet..

[45] Chen J., Ao L., Yang J. (2019). Long non-coding RNAs in diseases related to inflammation and immunity. Ann. Transl. Med..

[46] Tong C. (2017). Screening and Characterization of Exosomal miRNAs and lncRNAs as Biomarkers in Bovine Mastitis. Ph.D. Thesis.

[47] Tong C., Chen Q., Zhao L., Ma J., Ibeagha-Awemu E.M., Zhao X. (2017). Identification and characterization of long intergenic noncoding RNAs in bovine mammary glands. BMC Genom..

[48] Wang H., Wang X., Li X., Wang Q., Qing S., Zhang Y., Gao M.Q. (2019). A novel long non-coding RNA regulates the immune response in MAC-T cells and contributes to bovine mastitis. FEBS J..

[49] Ozdemir S., Altun S. (2020). Genome-wide analysis of mRNAs and lncRNAs in mycoplasma bovis infected and non-infected bovine mammary gland tissues. Mol. Cell Probes..

[50] Wang X., Su F., Yu X., Geng N., Li L., Wang R., Zhang M., Liu J., Liu Y., Han B. (2020). RNA-Seq whole transcriptome analysis of bovine mammary epithelial cells in response to intracellular *Staphylococcus aureus*. Front. Vet. Sci..

[51] Chen Y., Jing H., Chen M., Liang W., Yang J., Deng G., Guo M. (2021). Transcriptional profiling of exosomes derived from Staphylococcus aureus-infected bovine mammary epithelial cell line MAC-T by RNA-Seq analysis. Oxid. Med. Cell Longev..

[52] Wang J.P., Hu Q.C., Yang J., Luoreng Z.M., Wang X.P., Ma Y., Wei D.W. (2021). Differential expression profiles of lncRNA following LPS-induced inflammation in bovine mammary epithelial cells. Front. Vet. Sci..

[53] Brannan C.I., Dees E.C., Ingram R.S., Tilghman S.M. (1990). The product of the H19 gene may function as an RNA. Mol. Cell Biol..

[54] Sallam T., Sandhu J., Tontonoz P. (2018). Long noncoding RNA discovery in cardiovascular disease: Decoding form to function. Circ. Res..

[55] Sun Y., Zhong L., He X., Wang S., Lai Y., Wu W., Song H., Chen Y., Yang Y., Liao W. (2019). LncRNA H19 promotes vascular inflammation and abdominal aortic aneurysm formation by functioning as a competing endogenous RNA. J. Mol. Cell Cardiol..

[56] Müller V., Oliveira-Ferrer L., Steinbach B., Pantel K., Schwarzenbach H. (2019). Interplay of lncRNA H19/miR-675 and lncRNA NEAT1/miR-204 in breast cancer. Mol. Oncol..

[57] Singh N., Ramnarine V.R., Song J.H., Pandey R., Padi S., Nouri M., Olive V., Kobelev M., Okumura K., McCarthy D. (2021). The long noncoding RNA H19 regulates tumor plasticity in neuroendocrine prostate cancer. Nat. Commun..

[58] Yang W., Li X., Qi S., Li X., Zhou K., Qing S., Zhang Y., Gao M.Q. (2017). lncRNA H19 is involved in TGF-beta1-induced epithelial to mesenchymal transition in bovine epithelial cells through PI3K/AKT signaling pathway. PeerJ.

[59] Baulida J. (2017). Epithelial-to-mesenchymal transition transcription factors in cancer-associated fibroblasts. Mol. Oncol..

[60] Zeisberg M., Hanai J., Sugimoto H., Mammoto T., Charytan D., Strutz F., Kalluri R. (2003). BMP-7 counteracts TGF-beta1-induced epithelial-to-mesenchymal transition and reverses chronic renal injury. Nat. Med..

[61] Li X., Wang H., Zhang Y., Zhang J., Qi S., Zhang Y., Gao M.Q. (2019). Overexpression of lncRNA H19 changes basic characteristics and affects immune response of bovine mammary epithelial cells. PeerJ.

[62] Chen Q., Yang W., Wang X., Li X., Qi S., Zhang Y., Gao M.Q. (2017). TGF-beta1 induces EMT in bovine mammary epithelial cells through the TGFbeta1/Smad signaling pathway. Cell Physiol. Biochem..

[63] Loda A., Heard E. (2019). Xist RNA in action: Past, present, and future. PLoS Genet..

[64] Brown C.J., Ballabio A., Rupert J.L., Lafreniere R.G., Grompe M., Tonlorenzi R., Willard H.F. (1991). A gene from the region of the human X inactivation centre is expressed exclusively from the inactive X chromosome. Nature.

[65] Ma M., Pei Y., Wang X., Feng J., Zhang Y., Gao M.Q. (2019). LncRNA XIST mediates bovine mammary epithelial cell inflammatory response via NF-kappaB/NLRP3 inflammasome pathway. Cell Prolif..

[66] Dajon M., Iribarren K., Cremer I. (2017). Toll-like receptor stimulation in cancer: A pro- and anti-tumor double-edged sword. Immunobiology.

[67] Carpenter S., Fitzgerald K.A. (2018). Cytokines and long noncoding RNAs. Cold Spring Harb. Perspect. Biol..

[68] Hadjicharalambous M.R., Lindsay M.A. (2019). Long non-coding RNAs and the innate immune response. Noncoding RNA.

[69] Zhao G., Su Z., Song D., Mao Y., Mao X. (2016). The long noncoding RNA MALAT1 regulates the lipopolysaccharide-induced inflammatory response through its interaction with NF-kappaB. FEBS Lett..

[70] Wellnitz O., Arnold E.T., Lehmann M., Bruckmaier R.M. (2013). Short communication: Differential immunoglobulin transfer during mastitis challenge by pathogen-specific components. J. Dairy Sci..

[71] Wang X., Wang H., Zhang R., Li D., Gao M.Q. (2020). LRRC75A antisense lncRNA1 knockout attenuates inflammatory responses of bovine mammary epithelial cells. Int. J. Biol. Sci..

[72] Zhang X., Cheng Z., Wang L., Jiao B., Yang H., Wang X. (2019). MiR-21-3p centric regulatory network in dairy cow mammary epithelial cell proliferation. J. Agr. Food Chem..

[73] Yang B. (2019). Screening, Identification and Functional Studies of Long Non-Coding RNAs Differentially Expressed in Mammary Gland of Dairy Cows. Ph.D. Thesis.

[74] Tucker A.R., Salazar N.A., Ayoola A.O., Memili E., Thomas B.N., Morenikeji O.B. (2021). Regulatory network of miRNA, lncRNA, transcription factor and target immune response genes in bovine mastitis. Sci. Rep..

